# Sustainable Fish Meal-Free Diets for Gilthead Sea Bream (*Sparus aurata*): Integrated Biomarker Response to Assess the Effects on Growth Performance, Lipid Metabolism, Antioxidant Defense and Immunological Status

**DOI:** 10.3390/ani14152166

**Published:** 2024-07-25

**Authors:** Ana M. Fernandes, Josep Àlvar Calduch-Giner, Gabriella V. Pereira, Ana Teresa Gonçalves, Jorge Dias, Johan Johansen, Tomé Silva, Fernando Naya-Català, Carla Piazzon, Ariadna Sitjà-Bobadilla, Benjamin Costas, Luís E. C. Conceição, Jorge M. O. Fernandes, Jaume Pérez-Sánchez

**Affiliations:** 1 Sparos Lda, 8700-221 Olhão, Portugal; anafernandes@sparos.pt (A.M.F.); gabriellapereira@sparos.pt (G.V.P.); anagoncalves@sparos.pt (A.T.G.); jorgedias@sparos.pt (J.D.); tomesilva@sparos.pt (T.S.); luisconceicao@sparos.pt (L.E.C.C.); 2 Faculty of Biosciences and Aquaculture, Nord University, 8049 Bodø, Norway; jorge.m.fernandes@nord.no; 3 Institute of Aquaculture Torre de la Sal (IATS, CSIC), 12595 Ribera de Cabanes, Castellón, Spain; j.calduch@csic.es (J.À.C.-G.); fernando.naya@iats.csic.es (F.N.-C.); carla.piazzon@csic.es (C.P.);; 4 GreenCoLab—Associação Oceano Verde, Universidade do Algarve, 8005-139 Faro, Portugal; 5 Norwegian Institute of Bioeconomy Research (NIBIO), 1431 Ås, Norway; johan.johansen@nibio.no; 6 Interdisciplinary Centre of Marine and Environmental Research (CIIMAR), University of Porto, 4200-465 Porto, Portugal; bcostas@ciimar.up.pt; 7 School of Biomedicine and Biomedical Sciences (ICBAS), University of Porto, 4200-465 Porto, Portugal

**Keywords:** gilthead sea bream, sustainable aquaculture, novel ingredient combinations, hepatic lipogenesis, antioxidant defense, inflammatory status

## Abstract

**Simple Summary:**

The present study shows how different combinations of eco-efficient ingredients can be considered reliable solutions for European aquaculture production. For this purpose, three different formulations were designed and tested in a 77-day gilthead sea bream trial. All diets supported similar growth rates, but the diet without poultry meal and processed animal proteins as the main fish meal replacer (NOPAP diet) shaped better nutrient utilization with an overall improvement of biomarkers of physiological condition. Such an integrative approach contributes to better understanding of what could be the best and least-cost feed formulation for an effective balance of sustainability and aquaculture profitability in a scenario of intensive aquaculture production and limited availability of marine feed ingredients for fish production.

**Abstract:**

The growth of the aquaculture industry requires more sustainable and circular economy-driven aquafeed formulas. Thus, the goal of the present study was to assess in farmed gilthead sea bream (*Sparus aurata* L.) how different combinations of novel and conventional fish feed ingredients supported proper animal performance in terms of growth and physiological biomarkers of blood/liver/head kidney. A 77-day feeding trial was conducted with three experimental diets (PAP, with terrestrial processed animal protein from animal by-products; NOPAP, without processed animal protein from terrestrial animal by-products; MIX, a combination of alternative ingredients of PAP and NOPAP diets) and a commercial-type formulation (CTRL), and their effects on growth performance and markers of endocrine growth regulation, lipid metabolism, antioxidant defense and inflammatory condition were assessed at circulatory and tissue level (liver, head kidney). Growth performance was similar among all dietary treatments. However, fish fed the PAP diet displayed a lower feed conversion and protein efficiency, with intermediate values in MIX-fed fish. Such gradual variation in growth performance was supported by different biomarker signatures that delineated a lower risk of oxidation and inflammatory condition in NOPAP fish, in concurrence with an enhanced hepatic lipogenesis that did not represent a risk of lipoid liver degeneration.

## 1. Introduction

The contribution of aquaculture to total fisheries and aquaculture production reached 49.2% out of 178 million tons in 2020. From this total, 12% was used for purposes other than human consumption, namely the production of fish meal (FM) and fish oil (FO) [1]. Such feedstuffs have been used for a long time in marine aquaculture, not only because of their nutrition and digestibility value but also as a major source of n-3 long-chain polyunsaturated fatty acids (LC-PUFA), mainly eicosapentaenoic acid (EPA) and docosahexaenoic acid (DHA) [2,3]. However, both FM and FO are finite resources and with the increasing environmental and social concerns of consumers and society, continuous efforts have been made over the last two decades to reduce their inclusion level in marine fish feeds [4,5,6,7,8,9]. Common FM replacers are soybean [10,11,12] and pea proteins [13,14] among other plant protein-rich ingredients [15]. However, these ingredients include some anti-nutritional factors (e.g., protease inhibitors, lectins, phytic acid, saponins, phytoestrogens, antivitamins, allergens) that can negatively affect the overall fish performance [16,17]. Luckily, most of these drawback effects can be totally or partially alleviated by the use of feed additives [17,18,19,20,21,22], though their success and mode of action is largely shaped in European sea bass (*Dicentrarchus labrax*) [23] and gilthead sea bream (*Sparus aurata*) [24] by the genetic background.

According to the above findings, a practical diet based on a mix of plant proteins, insect proteins and single cell proteins (SCP), adequately supplemented with a commercial health promoter (Sanacore^®^ GM), preserved proper growth in gilthead sea bream without impairment of disease outcome when exposed to a myxosporean intestinal parasite, *Enteromyum leei* [25]. Insect meal is, in fact, a promising dietary protein source for aquatic and terrestrial livestock [26,27,28]. In particular, good performance results have been achieved in Mediterranean farmed fish with total or partial defatted insect meal, varying the FM replacement between 25% and 80% [29,30,31]. Thus far, the European Commission has approved the use of seven insect species in aquafeeds (Regulation 2017/893/EC). The materials of animal origin that can be used for farmed insect feeding are restricted (Regulation 2017/1017/EC), so manure, waste, or former foodstuffs containing meat, fish or food losses are excluded [27]. Like insect proteins, SCPs are becoming a highly promising dietary protein with encouraging results in main cultured farmed fish [32,33,34], including gilthead sea bream, in which the use of SCP as an FM replacer supported efficient growth and showed anti-inflammatory actions, linked to the reshaping of the gut microbiota composition [35]. The search for sustainable dietary proteins has also led to consideration of the use of processed animal proteins (PAP) from farm animals (blood, meat and bone, feathers) as an alternative ingredient in aquafeeds. These feedstuffs meet the phosphorus and amino acid fish requirements (lysine, sulfur amino acids, histidine and tryptophan), usually considered limiting in other dietary protein sources [31,36,37,38,39,40]. However, the consumer view of PAPs is largely negatively biased, though the use of PAPs from fish and non-ruminant animals (poultry and pigs) was permitted in aquafeeds since 2013 with strict traceability requirements and analytical controls (2013/56/EC).

FM usually contains 8–12% of fish oil and its gradual replacement in marine aquafeeds entails the use of other oil sources, such as micro-algae oil [41,42,43], fish oil by-products [44], and tunicate extracts [45], which are now recognized as strategic marine bio-resources with many potential applications [46]. As a result of the replacement strategies in aquafeeds, the inclusion of marine ingredients in the Norwegian salmon diet have gradually decreased from 90% in 1990 to 25% in 2016 [47,48,49]. Similar achievements have been made in gilthead sea bream, varying the inclusion level of marine feedstuffs from more than 50% at the beginning of this century [50] to less than 10% in plant-based diets, supporting maximum growth from early life stages to completion of sexual maturation in three-year old fish [51,52]. In any case, strategies for balancing sustainability and aquaculture profitability in aquafeeds composition continue to be a primary challenge, and least-cost feed formulations are approached as a standard method that minimizes feed costs while satisfying the threshold constraints of nutritional requirements [53]. In this way, the continuous updating of the functional assessment procedures that are moving towards cost-effective multi-omic and multi-tissue target approaches makes particular sense, including the study of the organism interaction with the environment from a holobiont perspective. Thus, as part of a global study, we considered different ingredient baskets to simultaneously fulfill, in a cost-effective manner, the nutritional requirements and technical constraints of gilthead sea bream farming, with an annual production of 282,000 tons in 2020 [1]. As a first outcome, we linked the use of PAP and NOPAP FM-free diets with changes in gut microbiota and host transcriptomics with a special focus at the local intestinal level [54]. The aim of the present study was to go further in the evaluation of these diets, with the addition of a new one with a combination of alternative ingredients from both diets, in terms of growth performance, and biochemical and gene expression landmarks of blood/liver/head kidney, including markers of endocrine growth regulation, lipid and energy metabolism, antioxidant defense and immunological status.

## 2. Materials and Methods

### 2.1. Ethics Statement

The feeding trial was conducted by trained scientists (following FELASA category C recommendations) and according to EU animal experimentation guidelines on the protection of animals used for scientific purposes (2010/63/EC) at the experimental facilities of RIASEARCH/SPAROS (Murtosa, Portugal) (approval number 13984).

### 2.2. Diets

Four extruded diets were formulated and manufactured by SPAROS Lda (Olhão, Portugal) to be isoenergetic, isoproteic and isolipidic, considering the nutritional requirements for gilthead sea bream juveniles (Table 1 and Table S1). The control (CTRL) diet had a formulation mimicking a commercial diet, with 20% FM and a high content of vegetable protein sources. The composition and ingredients origin of the other three diets were designed to facilitate aquaculture eco-intensification through increased circularity and resource utilization: (1) NOPAP—Circular economy-driven formula without PAPs from terrestrial animal by-products; (2) PAP—Circular economy-driven formula rich in terrestrial PAPs from animal by-products, and (3) MIX—Circular economy-driven formula with a combination of alternative ingredients of PAP and NOPAP diets. Both the PAP- and NOPAP-based feeds contained insect meal, fish by-products, and microbial and yeast biomasses as replacements for FM and vegetable protein sources. The PAP-based diet also comprised poultry meal, feather meal hydrolysate, and porcine blood meal as main protein sources. Alternatively, the NOPAP-diet included rapeseed and corn gluten meals, along with FM from by-products and several single cell protein ingredients, such as yeast meal, microbial protein meal, Spirulina and Chlorella meals as novel protein sources. FO was replaced in both the PAP and NOPAP diets by an aquaculture by-product (salmon oil), DHA-rich algae biomass (Schizochytrium) and rapeseed oil, maintaining the EPA+DHA content (% dry matter) above (CTRL, 1.7%) or close (NOPAP, 0.79%; MIX, 0.93%; PAP, 0.96%;) to the established requirements (0.90%) for juveniles of gilthead sea bream [55].

### 2.3. Experimental Setup and Sampling

Up to 880 gilthead sea bream juveniles of 55.9 ± 0.51 g (mean ± SD, initial body weight) from a commercial hatchery (SONRIONANSA, Santander, Spain) were distributed into 16 tanks of 500 L (55 fish per tank in quadruplicate tanks per diet). Water parameters were recorded daily. Average values for dissolved oxygen concentration were 5.8 ± 0.3 mg/L, temperature 22.0 ± 1.4 °C and salinity 18.36 g/L with a fixed 12:12 h photoperiod. During the trial, fish were hand-fed ad libitum three times a day and feed waste was collected to calculate the daily feed intake. At the end of the trial (77 days), 10 fish from each tank were sedated with anesthetic tricaine methane sulphonate (MS-222, 0.1 g/L, Sigma-Aldrich, St. Louis, MO, USA) to collect blood from the caudal vessel using heparinized syringes (10 units heparin mL, Sigma-Aldrich). Blood was then transferred to Eppendorf tubes and centrifuged at 10,000× *g* for 10 min and the plasma collected, frozen on dry ice, and stored at −80 °C for evaluation of humoral parameters. After blood collection, the fish were sacrificed by an overdose of the same anesthetic at a lethal dose (0.2 g/L, Sigma-Aldrich), and head kidney and liver were collected, immediately frozen on dry ice and stored at −80 °C. In the case of liver, the tissue was split in two equal parts for gene expression analysis and oxidative status biomarkers.

### 2.4. Zootechnical Measurements

The growth performance indicators used were the relative growth rate (RGR), feed conversion ratio (FCR) and protein efficiency ratio (PER). These parameters were calculated as previously described by [28,56]:

RGR (% weight increase per day) = 100 × (e ^([ln(final body weight (g)) − ln(initial body weight (g))]/number of feeding days)^ − 1)

FCR = Feed intake (g)/(final body weight (g) − initial body weight (g))

PER = Gain in weight (g)/protein intake (g)

### 2.5. Humoral Immune Parameters

Protease activity was quantified using the azocasein hydrolysis assay according to the method of [57] with minor alterations. Briefly, 10 µL of plasma was incubated with 100 µL of sodium bicarbonate buffer (5 mg/mL NaHCO_3_, pH 8.3, Sigma-Aldrich) and 125 µL of azocasein (20 mg/mL in NaHCO_3_, 5 mg/mL, pH 8.3, Sigma-Aldrich) for 24 h at room temperature in an orbital shaker (100 rpm). The reaction was stopped by adding 250 µL of 10% trichloroacetic acid (TCA) (100 mg/mL, Sigma-Aldrich). Then, the mixture was centrifuged at 6000× *g* for 5 min. The supernatants (100 μL) were transferred to a 96-well plate in duplicate containing 100 μL of 1 N NaOH (40 mg/mL, Sigma-Aldrich), and the optical density (OD) read at 450 nm using a Synergy HT microplate reader (Biotek, Winooski, VT, USA). Plasma was replaced by trypsin (5 mg/L, Sigma-Aldrich) as positive control, corresponding to 100% of protease activity, or by buffer as negative control equivalent to 0% activity. Antiprotease activity was determined according to the method described by [58] adapted by [59] and conducted in duplicate. Plasma bactericidal activity was measured according to [60], adapted by [59] using the bacteria *Photobacterium damselae* subsp. *piscicida* (Phdp), strain PP3. Phdp was kindly provided by Dr. Ana do Vale (Institute for Molecular and Cell Biology, University of Porto, Portugal) and isolated from yellowtail (*Seriola quinqueradiata*) by Dr Andrew C. Barnes (Marine Laboratory, Aberdeen, UK). PP3 draft genome has been deposited at GenBank/EMBL/DDBJ under the GenBank accession number SRHT00000000, BioProject accession number PRJNA529570, and BioSample accession number SAMN11269591. Bacteria were routinely cultured at 22 °C in tryptic soy broth (TSB) or tryptic soy agar (TSA) (Difco, Le Pont de Claix, Francia), supplemented with NaCl to a final concentration of 1% (*w*/*v*) (TSB-1 and TSA-1, respectively) and stored at −70 °C in TSB-1 supplemented with 15% (*v*/*v*) glycerol. To prepare the inoculum for bactericidal activity, stocked bacteria were cultured for 48 h at 22 °C on TSA-1 and then inoculated into TSB-1 and cultured overnight at the same temperature, with continuous shaking. Exponentially growing bacteria were resuspended in sterile HBSS and adjusted to 1 × 10^6^ cfu/mL. Plating serial dilutions of the suspensions onto TSA-1 plates and counting the number of cfu following incubation at 22 °C confirmed the bacterial concentration of the inoculum.

Total plasma immunoglobulin M (IgM) levels were evaluated using the enzyme-linked immunosorbent assay (ELISA) described by [61] with minor alterations. First, 100 μL of plasma diluted 1:100 in Na_2_CO_3_ (50 mM, pH 9.6, Sigma-Aldrich) was placed in each well of a flat-bottomed 96-well plate in triplicate. Then, 100 μL of Na_2_CO_3_ 50 mM buffer was used as a negative control. Samples (antigen) were allowed to adhere to the plate at room temperature for 1 h. Thereafter, the sample was removed; 300 μL per well of blocking buffer (5% low-fat milk in T-TBS (0.1% Tween 20, Sigma-Aldrich)) was added and the plate was incubated for one hour at room temperature. The blocking buffer was removed, and the plate was washed three times with T-TBS. After this, 100 μL per well of diluted (1:200 in blocking buffer) anti-seabream IgM monoclonal antibody (Aquatic Diagnostics Ltd., Stirling, Scotland, UK) was added and incubated for 1 h. After removing the antibody, the plate was washed three times with T-TBS and 100 μL per well of diluted (1:1000 in blocking buffer) mouse secondary antibody (Aquatic Diagnostics Ltd.) was added and incubated for 1 h. Then, the plate was washed three times with T-TBS. Finally, 100 μL per well of TMB substrate solution for ELISA (Thermo Scientific, Bremen, Germany) was added and incubated for 5 min. The reaction was stopped with 100 μL of H_2_SO_4_ per well (2 M, Sigma-Aldrich), and the OD was read at 450 nm on the Synergy HT microplate reader (Biotek).

### 2.6. Oxidative Status

Liver samples were thawed and homogenized (1:10) in phosphate buffer 0.1 M (pH 7.4) using the Precellys evolution tissue lyser homogenizer (Bertin Technologies, Montigny-le-Bretonneux, France). One aliquot of tissue homogenate was used to determine the extent of endogenous lipid peroxidation (LPO) by measuring thiobarbituric acid-reactive species (TBARS) as suggested by [62]. To prevent artifactual peroxidation, butylhydroxytoluene (BHT 0.2 mM) was added to the aliquot. Briefly, 1 mL of 100% trichloroacetic acid and 1 mL of 0.73% thiobarbituric acid solution (in Tris–HCl 60 mM pH 7.4 with DTPA 0.1 mM) was added to 0.2 mL of liver homogenate. After incubation at 100 °C for 60 min, the solution was centrifuged at 12,000× *g* for 5 min and LPO levels were determined at 535 nm. The remaining tissue homogenate was centrifuged for 20 min at 10,000× *g* (4 °C) to obtain the post-mitochondrial supernatant fraction (PMS). Total proteins in homogenates were measured by using a Pierce™ BCA Protein Assay Kit, as described by the manufacturer.

Catalase (CAT) activity was determined in PMS by measuring substrate (H_2_O_2_) consumption at 240 nm according to [63] adapted to microplate. Briefly, in a microplate well, 0.140 mL of phosphate buffer (0.05 M, pH 7.0) and 0.150 mL H_2_O_2_ solution (30 mM in phosphate buffer 0.05 M, pH 7.0) were added to 0.01 mL of liver PMS (0.7 mg/mL total protein). Enzymatic activity was determined in a Synergy HT microplate reader (BioTek) reading the optical density at 240 nm for 2 min every 15 sec interval.

### 2.7. PCR-Array

Total RNA from liver and head kidney from 10 to 12 fish per diet, was extracted using the MagMax-96 total RNA isolation kit (Life Technologies, Carlsbad, CA, USA). The RNA yield was higher than 3.5 µg with absorbance measures (A_260/280_) of 1.9–2.1 on a Nanodrop 2000c (Thermo Scientific, Bremen, Germany). Synthesis of cDNA was performed with the High-Capacity cDNA Archive Kit (Applied Biosystems, Foster City, CA, USA), using random decamers and 500 ng of total RNA in a final volume of 100 µL. Reverse transcription (RT) reactions were incubated for 10 min at 25 °C and 2 h at 37 °C. Negative control reactions were run without RT. Two different 96-well PCR-array layouts were designed for the simultaneous profiling of a panel of 42 (liver) and 29 (head kidney) genes (Tables S2 and S3). Such arrays included markers of growth performance (9), lipid metabolism (15), energy metabolism (6), antioxidant defense (9) intracellular proteolysis (3), immune cell communication (11), adaptive immunity (2), acute phase response and proteolytic activity (4), T-cell and monocyte/macrophage cells (7) and pathogen recognition receptors (5). Real-time quantitative PCR reactions were performed using an iCycler IQ Real-time Detection System (Bio-Rad, Hercules, CA, USA). Diluted RT reactions were used for qPCR assays in a 25 µL volume with an SYBR Green Master Mix (Bio-Rad) and specific primers at a final concentration of 0.9 µM (Tables S4 and S5). The program used for PCR amplification included an initial denaturation step at 95 °C for 3 min, followed by 40 cycles of denaturation for 15 s at 95 °C and annealing/extension for 60 s at 60 °C. All the pipetting operations were made by means of an EpMotion 5070 Liquid Handling Robot (Eppendorf, Hamburg, Germany) to improve data reproducibility. The efficiency of PCRs (≥92%) was checked, and the specificity of reactions was verified by analysis of melting curves (ramping rates of 0.5 °C/10 s over a temperature range of 55–95 °C). Fluorescence data acquired during the extension phase were normalized by the delta-delta Ct method [64] using beta-actin as a housekeeping gene due to its stability among different experimental conditions—average CT values among experimental groups varied less than 0.2 for a given tissue. For multigene analysis, all values in liver were referenced to the expression levels of cyp7a1 in CTRL fish with an assigned value of 1.0. For head kidney, expression values were referenced to those of il10 in CTRL fish with an assigned value of 1.0.

### 2.8. Statistical Analysis

Statistical analyses were performed using the package IBM SPSS Statistics 26 for Windows, with the level of significance set at *p* ≤ 0.05. Data growth performance, biochemical parameters and relative gene expression were checked for data normality (Kolmogorov–Smirnova test) and analyzed by one-way ANOVA followed by Tukey post hoc test. The fold-changes in gene expression between experimental and control groups were analyzed by Student *t*-test. Z Genesis software v9.1 [65] was used to generate heatmaps of data gene expression.

## 3. Results

### 3.1. Growth Performance

Overall growth performance after 77 days was similar in all dietary groups, with no significant differences in terms of final body weight or weight gain between fish fed the CTRL diet and those fed the experimental diets (Table 2). Nevertheless, feed conversion and protein efficiency presented significant differences (*p* < 0.05) among dietary treatments. Fish fed CTRL diet showed a significantly lower FCR (1.40) than those fed with the new formulated diets, that ranged between 1.48 (NOPAP) and 1.62 (PAP). The most efficient PER was that of fish fed CTRL (1.57) and NOPAP (1.50) diets, significantly higher than those fed MIX (1.39) and PAP (1.33) diets.

### 3.2. Humoral Parameters and Oxidative Status

Plasma protease and bactericidal activities were not altered by dietary treatments (Table 3). The highest values of anti-protease activity were measured in gilthead sea bream fed PAP and CTRL diets (81.13–81.33%) whereas that found in fish fed the MIX diet was significantly (*p* < 0.05) lower (78.96%). Plasma IgM levels ranged from 0.27 (PAP) to 0.35 (MIX), with a significant difference between these two dietary groups.

Hepatic CAT activity and TBARS levels are displayed in Table 4. The antioxidant enzyme CAT presented a significant lower activity (*p* < 0.0001) between fish fed the CTRL diet and those fed the tested feed formulations. Hepatic CAT activity varied between 28.67 and 46.37 U/mg protein. Lipid peroxidation was not affected by the different formulations.

### 3.3. Liver and Head Kidney Gene Expression Profiling

**Figure 1:**
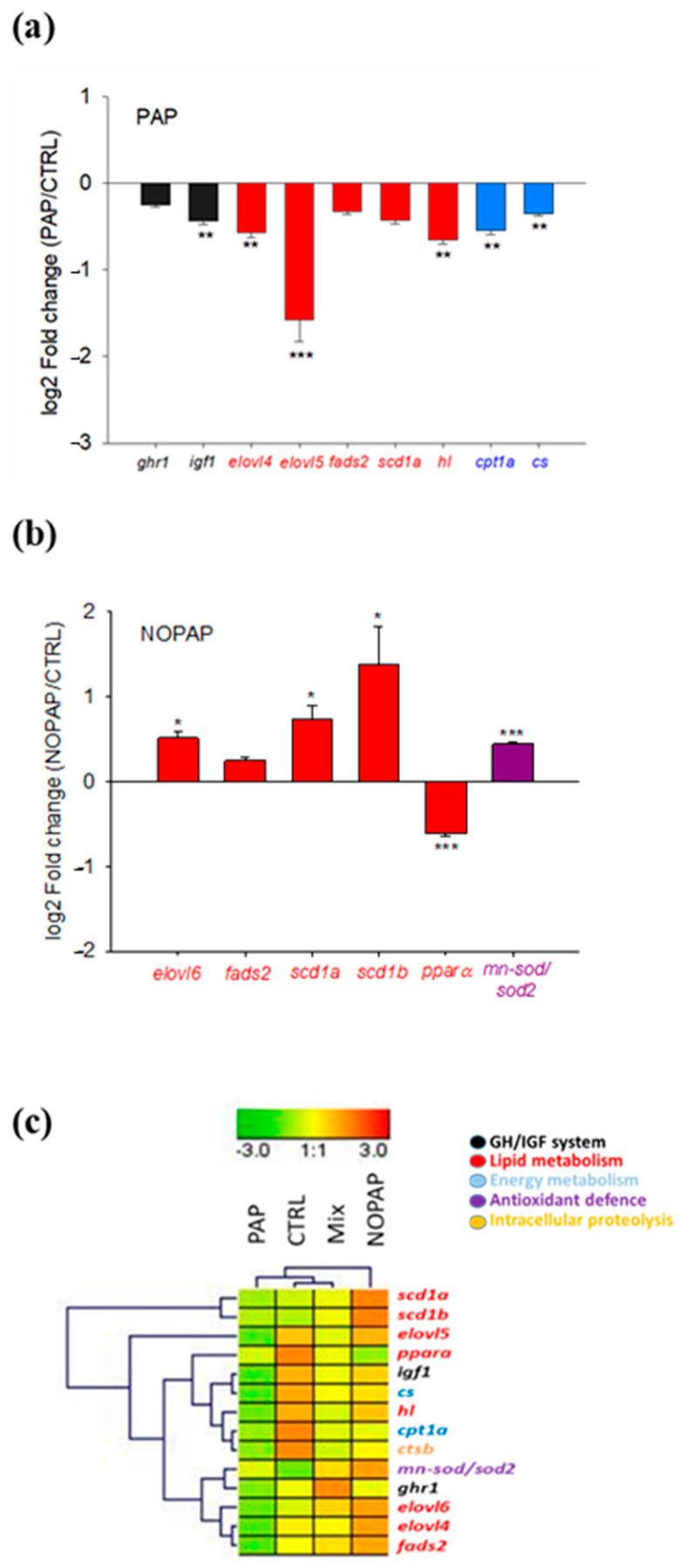
Expression fold-change (experimental/CTRL) of relevant hepatic genes in PAP (**a**) and NOPAP (**b**) groups, according to their fold-change values and statistical significance (Student-*t* test). Values are the mean ± SEM of 10–12 fish. Asterisks indicate statistically significant differences (*, *p* < 0.05; **, *p* < 0.01; ***, *p* < 0.001). (**c**) Heatmap of liver gene expression profile of most nutritionally regulated genes.

Eleven out of 42 genes analyzed in the liver were significantly (*p* < 0.05) regulated by dietary intervention in at least one dietary group (Table 5). Within these genes, there was a set of markers of growth performance (insulin-like growth factor-1, *igf1*), antioxidant defense (superoxide dismutase [Mn], *mn-sod/sod2*), and lipid and energy metabolism, including fatty acid desaturases (stearoyl-CoA desaturase 1a, *scd1a;* stearoyl-CoA desaturase 1b, *scd1b*), fatty acid elongases (elongation of very-long-chain fatty acids 4, *elovl4*; elongation of very-long-chain fatty acids 5, *elovl5*; elongation of very-long-chain fatty acids 6, *elovl6*), lipases (hepatic lipase, *hl*), transcription factors (peroxisome proliferator-activated receptor α, *pparα*), and mitochondrial enzymes (carnitine palmitoyltransferase 1A, *cpt1a*; citrate synthase, *cs*). Overall, fish fed the PAP diet showed a down-regulated response in comparison to CTRL fish that was statistically significant (*p* < 0.05) for *igf1*, *elovl4*, *elovl5*, *hl*, *cpt1a* and *cs* (Figure 1a). A similar trend, but not statistically significant, was found for growth hormone receptors (*ghr1, ghr2*), fatty acid desaturase 2 (*fads2*) and *scd1a*. Conversely, with the exception of *pparα*, the overall trend for NOPAP fish was an up-regulated response that was statistically significant for *scd1a*, *scd1b* and *elovl6*. A similar, but less evident, trend was found for *fads2*, while *elovl5*, *hl* and *cs* remained almost invariant in comparison to CTRL fish (Figure 1b). MIX fish often shared intermediate gene expression levels between PAP and NOPAP fish, and the clustering heatmap comprising nutritionally regulated genes (with significance set at *p* < 0.1) joint-clustered CTRL and MIX fish also embracing PAP fish, while NOPAP fish appeared in a separated branch (Figure 1c).

**Figure 2:**
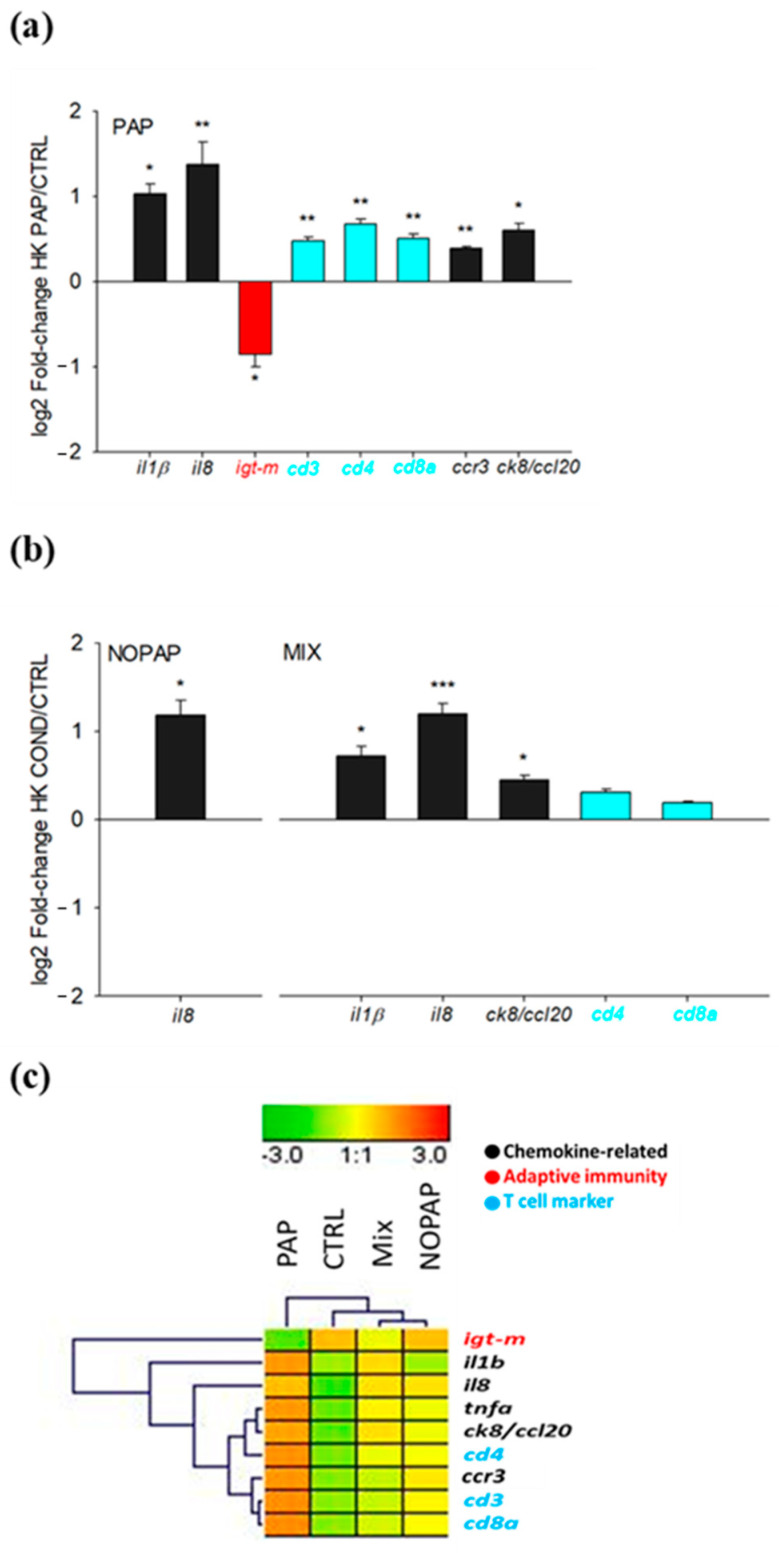
Expression fold-change (experimental/CTRL) of relevant head kidney genes markers in PAP (**a**) and NOPAP and MIX (**b**) groups, according to their fold-change values and statistical significance (Student-*t* test). Values are the mean ± SEM of 10–12 fish. Asterisks indicate statistically significant differences (*, *p* < 0.05; **, *p* < 0.01; ***, *p* < 0.001). (**c**) Heatmap of head kidney gene expression profile of differentially expressed genes.

Nine out of 29 genes analyzed in the head kidney showed a significant (*p* < 0.05) difference by dietary treatment (Table 6). As a general trend, interleukin-8 (*il8*) was up-regulated in all experimental groups. In PAP fish, this pro-inflammatory pattern was reinforced by the up-regulated expression of interleukin-1 beta (*il1β*), tumor necrosis factor-alpha (*tnfα*), C-C chemokine CK8/C-C motif chemokine 20 (*ck8/ccl20*)), and C-C chemokine receptor type 3 (*ccr3*). The same pattern was found for T-cell markers, including cluster of differentiation 3 zeta chain (*cd3*), cluster of differentiation 4 (*cd4*), and cluster of differentiation 8 alpha (*cd8α*), while the expression of *igt-m* was consistently down-regulated in this group of fish (Figure 2a). Likewise, MIX fish showed a pro-inflammatory expression profile that in addition to *il8* was extensive to *il1β* and *ck8*, and to a lower extent to *cd4* and *cd8a* (Figure 2b). As a result of all this, the clustering heatmap of these differentially expressed genes joint-clustered MIX and NOPAP fish with CTRL fish, with remaining NOPAP closer to CTRL fish, while PAP persisted as a separate group (Figure 2c).

## 4. Discussion

Many efforts and much progress have been made over the last 20 years for the replacement of FM and FO in aquafeeds of many species, including salmonid and non-salmonid fish [6,7,9,21]. In the present study, we did not focus on any specific ingredients, but the achieved outcomes provide new insights and criteria to evaluate the sustainability of different FM-free diet formulations according to the principles of circular economy, which aimed to contribute to zero waste while meeting the theoretical nutrient requirements of the species. As a first item, it was conclusively shown from our results that all the experimental diets (NOPAP, MIX, PAP) were able to support similar growth in comparison to the CTRL diet. Moreover, the lack of differences in voluntary feed intake pointed out no palatability issues, usually associated with a high replacement of FM and FO in marine fish feeds [66,67]. Despite this, it must be noted that fish fed the NOPAP diet displayed the best FCR and PER in comparison to the other two experimental groups (PAP, MIX), which would indicate improved nutrient absorption and/or utilization. This observation was reinforced by the finding that the hepatic expression of *igf1*, a well-known marker of growth performance in fish [68,69], did not differ from that found in CTRL fish, whereas its expression was significantly down-regulated in fish fed the PAP diet. The same trend, though it was not significant, was found in PAP fish for *ghr1*, and to a lower extent for the duplicated *ghr2*, the two main mediators of the Igf action in fish with a prompted tissue-specific differentiation during early life in the case of gilthead sea bream [70]. Conversely, it must be noted that the expression pattern of the Gh/Igf system in MIX fish mostly mimicked that found in CTRL and NOPAP fish, which is perhaps indicative that only minor growth-disturbing effects might occur over time with the use of both NOPAP and MIX diet formulations.

Lipoid liver degeneration is considered a frequent metabolic disturbance in gilthead sea bream fed practical or semi-purified diets formulated to be deficient in n-3 LC-PUFA [71,72]. This is in fact driven by the up-regulation of two key lipogenic enzymes, *scd1* that catalyzes the rate-limiting step in the formation of monounsaturated fatty acids (MUFAs), specifically oleate and palmitoleate from stearoyl-CoA and palmitoyl-CoA, respectively; and *elovl6* that acts to convert saturated and MUFA C16 fatty acids into C18 species [73]. In the present study, the three experimental diets were formulated to keep the content of EPA+DHA close to the nutrient requirements of gilthead sea bream, but the final content varied between 0.79% (NOPAP diet) and 0.96% (PAP diet). In agreement with this, the two gilthead sea bream isoforms of *scd1*, first characterized in [74], achieved the highest expression level in NOPAP fish with the reduction in the dietary EPA+DHA content. The same but less prominent trend was found with *elovl6*, which is viewed as a co-regulated gene expression to increase the unsaturation fatty acid index of cell membranes in the absence of a surplus of dietary LC-PUFA, as was the case of fish fed the CTRL diet (EPA+DHA, 1.7%). The yang of this physiological regulation is the enhanced expression or lipogenic enzymes for the novo synthesis of MUFAs, while the yin is that deregulated lipogenesis can progress to fatty liver disease [75,76]. However, NOPAP fish did not seem to face a real risk of hepatic steatosis in the absence of an enhanced expression of *elovl5*, a main component of the negative feedback loop of lipogenesis that acts to trigger the activation of triglyceride catabolism [77,78]. The precise regulation of this negative feedback loop remains to be elucidated in fish, though it appears that it can be modulated by a number of feed additives (e.g., spices, bile salts), with a recently reported anti-steatosis effect in gilthead sea bream [79,80,81].

Another key actor in the regulation of lipid metabolism is the gut microbiota, as it can modulate a wide range of host physiological processes from intermediary metabolism to cognitive function [82,83,84]. Certainly, some gut bacteria taxa, such as *Serratia* and *Brevinema*, are related to the production of short-chain fatty acids, and their higher abundance in NOPAP fish was positively correlated with the hepatic expression of *scd1* and *elovl6* [54]. Such a metabolic feature would serve to ensure the provision of lipogenic substrates to address increased production of MUFAs. Otherwise, it is well known that short-chain fatty acids exert both anti-inflammatory and antioxidant actions [85,86], and their increased production in the gut of NOPAP fish could mediate, at least in part, the up-regulation of hepatic antioxidant markers (*mn-sod/sod2*), in concurrence with the anti-inflammatory gene expression pattern of the head kidney (see below). This role can also be attributed to the antioxidant/anti-inflammatory properties of micro-algae (*Spirulina* and *Chlorella*) [87], which were added as FM replacers in the NOPAP and MIX diets. In the end, no changes in hepatic LPO damage were found among fish groups despite the higher susceptibility of PUFAs and, thereby, CTRL fish to lipid peroxidation [88], which was apparently counteracted among other measures by the increased activity of hepatic catalase. Therefore, it appears that CTRL and experimental fish achieved different antioxidant and gene expression trade-offs. In PAP fish, such regulated expression rendered the down-regulation of several markers of Gh/Igf system and lipid metabolism, including fatty elongases (*eolvl4, elovl5*) and desaturases (*fads2*), mitochondrial fatty acid transporters (*cpt1a*), lipoprotein lipases (*hl*), and markers of intact mitochondria (*cs*). As a result of this, the number of differentially regulated genes was highest in PAP fish, but the magnitude of change was relatively low and the resulting heatmap of hepatic gene expression embraced together CTRL and PAP fish, and MIX fish in a second branch, with the remaining NOPAP fish as an outlier.

Regarding immune parameters, the consistent up-regulation of pro-inflammatory genes (*il1β, il8, tnfα*), chemokine receptors (*ccr3, ck8/ccl20*) and T-cell markers (*cd3, cd4, cd8α*) in the head kidney of fish fed PAP diet suggested an inflammatory phenotype in this dietary group of animals. The effect of MIX diet on head kidney was again intermediate between that of PAP and NOPAP diets, and the alleviation of the pro-inflammatory pattern of NOPAP-fed fish (with *il8* as the only differentially regulated marker) was also consistent with the best performance of this dietary group. A similar expression trend was found for major cytokines involved in innate immunity, namely, *tnfα*, *il1β* and *il8* [89], with higher values in PAP fish and those of NOPAP fish closer to control. The role of these cytokines in initiating inflammatory events involves a cascade initiated by Tnf-α leading to the downstream expression of *il1β* and chemokines such as *il8*. Il1β possesses numerous functions, including the activation of immune cells and the regulation of metabolism [90]. Cytokines *il8* and *tnfα* can promote inflammatory reactions and recruit leukocytes in inflammation areas [91]. In our experimental model, the most responsive immune marker was the pro-inflammatory cytokine *il8*, a potent attractant of neutrophils in mammals and fish [92,93], that was significantly up-regulated in comparison to CTRL in the head kidney of the three experimental dietary groups. This result agrees with the up-regulation at gut level in response to PAP and NOPAP feeds [54] and reinforces the role of *il8* as a sensitive marker of inflammation in gilthead sea bream. The use of PAP diet also evidenced a down-regulation in head kidney expression of the membrane-bound form of immunoglobulin T (*igt-m*), widely used as a marker of mucosal immunocompetence [94,95], which may be indicative of an impaired immune response at the mucosal level. By contrast, *igm* expression or plasma levels did not follow the same regulation trend, a fact that agrees with other studies that suggested differential regulatory mechanisms for *igt-m* and *igm* in gilthead sea bream [54,94]. The overall head kidney expression profile clustered together MIX and NOPAP fish as closer to CTRL in the heatmap, with PAP fish as the most distinct group.

## 5. Conclusions

Similar growth rates were attained with the different gilthead sea bream feed formulations tested, though fish fed the diet with a high content of PAP-derived ingredients evidenced worse performance in terms of FCR and PER. Biomarker gene expression profiling also highlighted some physiological constraints, not only in growth, but also in pro-inflammatory markers. Conversely, the NOPAP diet emerged as the most favorable solution on the basis of growth performance, and antioxidant and anti-inflammatory markers, with intermediate values for fish fed the MIX diet. Differences in hepatic lipogenesis arose among dietary groups, but they did not have an impact on fish performance or health, though the routine monitoring of possible disturbances on lipid metabolism and inflammatory issues requires a special survey through all the production cycle. Ultimately, the suitability of each composition should be considered taking into account not only the expected animal performance, but also the scarcity and market value of traditional fish feed ingredients.

## Figures and Tables

**Table 1:** Ingredients and chemical composition of the experimental diets.

Ingredients (%)	CTRL	NOPAP	MIX	PAP
Fishmeal LT70	20.00			
Fishmeal 60 (by-products)		5.00		
Fish hydrolysate (by-products)		5.00	5.00	5.00
Insect meal		5.00	10.00	5.00
Microbial protein meal		5.00	10.00	5.00
Yeast protein meal		2.50	2.50	2.50
Feather meal hydrolysate			5.00	5.00
Porcine blood meal			3.00	3.00
Poultry meal 65	5.00		10.00	20.00
Microalgae meal (*Spirulina*)		5.00	5.00	
Microalgae meal (*Chlorella*)		0.50	0.50	
Soy protein concentrate	9.00			
Pea protein concentrate		4.10		
Wheat gluten	4.00	4.00		
Corn gluten meal	10.00	15.00	1.40	4.50
Soybean meal 48	12.00			
Rapeseed meal	4.00	11.50	7.00	5.70
Wheat meal	8.47			
Pea starch	3.00	7.90	9.00	6.00
Yellow peas	6.20	3.00	7.03	14.58
Fish oil	6.00			
Salmon oil		3.00	3.00	3.00
DHA-rich algae (*Schizochytrium*)		3.20	3.70	3.60
Rapeseed oil	8.26	8.50	6.30	6.00
Rapeseed lecithin	0.60	1.00	1.00	1.00
Vitamin and mineral premix *	1.00	1.00	1.00	1.00
Vitamin C (35%)	0.10	0.10	0.10	0.10
Brewer’s yeast		4.00	4.00	4.00
Macroalgae MIX		2.00	2.00	2.00
Antioxidant **	0.20	0.20	0.20	0.20
Sodium propionate	0.10	0.10	0.10	0.10
Monocalcium phosphate	2.00	2.50	2.20	1.90
L-Tryptophan	0.05	0.18	0.15	0.15
DL-Methionine		0.20	0.30	0.15
L-Taurine		0.50	0.50	0.50
Yttrium oxide	0.02	0.02	0.02	0.02
***Diet composition (as feed basis*) **				
Dry matter (%)	94.11	93.64	93.47	93.23
Ash (%)	8.47	7.19	8.05	8.35
Crude Protein (%)	44.20	44.88	45.30	44.71
Crude Lipid (%)	17.84	17.62	16.41	16.29
EPA + DHA (%)	1.70	0.79	0.93	0.96
Gross Energy (KJ/g)	21.50	21.90	21.20	20.65
NPE (MJ/kg feed)	3.30	3.30	3.50	3.40
PE ratio (MJ Gross Energy/g Crude Protein)	21.2	21.1	21.0	21.1

* In all diets, the inclusion of 1% of the vitamin and mineral premix contributed to an additional supply of the following micronutrients: Vitamins (IU or mg·kg^−1^ diet): DL-alpha tocopherol acetate, 255 mg; sodium menadione bisulphate, 10 mg; retinyl acetate, 26,000 IU; DL-cholecalciferol, 2500 IU; thiamine, 2 mg; riboflavin, 9 mg; pyridoxine, 5 mg; cyanocobalamin, 0.5 mg; nicotinic acid, 25 mg; folic acid, 4 mg; L-ascorbic acid monophosphate, 80 mg; inositol, 17.5 mg; biotin, 0.2 mg; calcium panthotenate, 60 mg; choline chloride, 1960 mg. Minerals (g or mg·kg^−1^ diet): copper sulphate, 8.25 mg; ferric sulphate, 68 mg; potassium iodide, 0.7 mg; manganese oxide, 35 mg; organic selenium, 0.01 mg; zinc sulphate, 123 mg; calcium carbonate, 1.5 g; excipient wheat middlings. ** VERDILOX (Kemin Europe NV, Herentals, Belgium).

**Table 2:** Growth performance of fish fed with four different diets. Values are the mean ± SD (*n* = 4). *p*-values from one-way ANOVA. Tukey post hoc test was used to identify differences between diet groups. Different lowercase letters indicate significant (*p* ≤ 0.05) differences between diet groups.

	CTRL	NOPAP	MIX	PAP	*p*-Value
IBW **^1^** (g)	56.22 ± 0.80	55.81 ± 0.33	55.91 ± 0.53	55.80 ± 0.35	0.713
FBW **^2^** (g)	136.69 ± 3.27	134.63 ± 6.35	132.04 ± 0.85	128.43 ± 3.88	0.070
Weight gain (g)	80.47 ± 2.88	78.82 ± 6.48	76.13 ± 0.77	72.63 ± 4.20	0.090
RGR **^3^** (% BW/d)	1.16 ± 0.03	1.15 ± 0.07	1.12 ± 0.01	1.09 ± 0.05	0.104
VFI **^4^** (%/day)	1.63 ± 0.06	1.70 ± 0.11	1.76 ± 0.03	1.76 ± 0.07	0.082
PER **^5^**	1.57 ± 0.01 ^a^	1.50 ± 0.03 ^a^	1.39 ± 0.04 ^b^	1.33 ± 0.01 ^b^	**<0.0001**
FCR **^6^**	1.40 ± 0.02 ^a^	1.48 ± 0.03 ^b^	1.56 ± 0.02 ^c^	1.62 ± 0.02 ^d^	**<0.0001**
Survival (%)	100 ± 0.00	100 ± 0.00	100 ± 0.00	100 ± 0.00	

^1^ IBW: Initial mean body weight; ^2^ FBW: Final mean body weight; ^3^ RGR: Relative growth rate; ^4^ VFI: Voluntary feed intake; ^5^ PER: Protein efficiency ratio; ^6^ FCR: Feed conversion ratio.

**Table 3:** Humoral parameters in fish fed with four different diets. Values are the mean ± SD (*n* = 40). *p*-values from one-way ANOVA. Tukey post hoc test was used to identify differences between diet groups. Different lowercase letters indicate significant (*p* ≤ 0.05) differences between diet groups.

	CTRL	NOPAP	MIX	PAP	*p*-Value
Protease (%)	7.27 ± 3.07	8.02 ± 3.01	7.69 ± 3.52	7.73 ± 3.35	0.783
Anti-protease (%)	81.33 ± 2.07 ^a^	79.86 ± 3.60 ^ab^	78.96 ± 5.31 ^b^	81.13 ± 1.40 ^a^	**0.008**
IgM (absorbance)	0.29 ± 0.08 ^ab^	0.33 ± 0.16 ^ab^	0.35 ± 0.11 ^a^	0.27 ± 0.14 ^b^	**0.031**
Bactericidal activity (%)	30.94 ± 7.48	35.69 ± 18.91	29.47 ± 6.06	31.39 ± 6.70	0.080

**Table 4:** Oxidative status biomarkers in fish fed with four different diets. Values are the mean ± SD (*n* = 40). *p*-values from one-way ANOVA. Tukey post hoc test was used to identify differences between diet groups. Different lowercase letters indicate significant (*p* ≤ 0.05) differences between diet groups.

	CTRL	NOPAP	MIX	PAP	*p*-Value
Catalase (U/mg protein)	46.37 ± 9.35 ^a^	36.42 ± 24.16 ^b^	28.67 ± 10.71 ^b^	32.05 ± 15.19 ^b^	**<0.0001**
**TBARS** (nmol/g)	12.81 ± 1.25	13.12 ± 1.42	13.54 ± 2.15	12.68 ± 0.98	0.081

**Table 5:** Relative gene expression of hepatic genes in juvenile fish fed experimental diets. Gene complete names and functions can be found in Table S2. Values are the mean ± SEM of 10–12 fish. *p*-values from one-way ANOVA. Tukey post hoc test was used to identify differences between diet groups. Different lowercase letters stand for significant (*p* ≤ 0.05) differences between diet groups. All data values are in reference to the expression level of cyp7a1 of fish fed CTRL diet with an arbitrary assigned value of 1.

	CTRL	NOPAP	MIX	PAP	*p*-Value
*ghr1*	2.24 ± 0.25	2.23 ± 0.20	2.56 ± 0.26	1.92 ± 0.18	0.320
*ghr2*	0.97 ± 0.07	0.98 ± 0.11	0.82 ± 0.10	0.81 ± 0.11	0.470
*igf1*	7.82 ± 0.83 ^a^	7.47 ± 0.76 ^a^	6.88 ± 0.61 ^ab^	5.80 ± 0.53 ^b^	**0.040**
*igf2*	2.77 ± 0.43	2.64 ± 0.62	2.42 ± 0.25	2.23 ± 0.40	0.836
*igfbp1a*	0.05 ± 0.01	0.04 ± 0.00	0.05 ± 0.01	0.04 ± 0.01	0.118
*igfbp1b*	6.09 ± 1.59	5.02 ± 1.62	4.60 ± 0.84	5.19 ± 1.11	0.881
*igfbp2a*	1.52 ± 0.13	1.49 ± 0.11	1.42 ± 0.15	1.36 ± 0.11	0.802
*igfbp2b*	1.67 ± 0.17	2.03 ± 0.14	1.68 ± 0.15	1.54 ± 0.11	0.300
*igfbp4*	0.58 ± 0.05	0.64 ± 0.04	0.69 ± 0.06	0.59 ± 0.04	0.417
*elovl1*	7.90 ± 0.49	7.82 ± 0.58	7.01 ± 0.52	6.66 ± 0.34	0.252
*elovl4*	0.18 ± 0.02 ^a^	0.19 ± 0.02 ^a^	0.16 ± 0.02 ^ab^	0.12 ± 0.01 ^b^	**0.036**
*elovl5*	2.53 ± 0.59 ^a^	2.49 ± 0.66 ^a^	1.58 ± 0.39 ^ab^	0.81 ± 0.14 ^b^	**0.022**
*elovl6*	1.71 ± 0.28 ^ab^	2.44 ± 0.40 ^a^	2.14 ± 0.52 ^ab^	1.32 ± 0.19 ^b^	**0.050**
*fads2*	2.04 ± 0.34	2.42 ± 0.37	2.18 ± 0.46	1.60 ± 0.17	0.417
*scd1a*	0.16 ± 0.02 ^ab^	0.27 ± 0.06 ^a^	0.20 ± 0.04 ^ab^	0.12 ± 0.01 ^b^	**0.044**
*scd1b*	0.32 ± 0.06 ^a^	0.59 ± 0.13 ^b^	0.53 ± 0.11 ^ab^	0.32 ± 0.10 ^a^	**0.049**
*hl*	7.93 ± 0.62 ^a^	7.61 ± 0.65 ^a^	6.24 ± 0.59 ^ab^	5.25 ± 0.44 ^b^	**0.007**
*atgl*	0.47 ± 0.11	0.42 ± 0.09	0.41 ± 0.06	0.60 ± 0.20	0.688
*lpl*	8.09 ± 1.26	7.49 ± 0.85	8.63 ± 0.91	7.58 ± 0.70	0.823
*pla2g6*	0.11 ± 0.01	0.13 ± 0.01	0.13 ± 0.01	0.11 ± 0.01	0.113
*cyp7a1*	1.12 ± 0.18	1.33 ± 0.18	1.29 ± 0.16	1.45 ± 0.22	0.653
*pparα*	2.22 ± 0.18 ^a^	1.46 ± 0.09 ^b^	1.69 ± 0.14 ^ab^	1.61 ± 0.17 ^ab^	**0.005**
*pparβ*	0.69 ± 0.09	0.80 ± 0.06	0.82 ± 0.08	0.76 ± 0.10	0.694
*pparγ*	0.29 ± 0.02	0.32 ± 0.02	0.28 ± 0.02	0.27 ± 0.02	0.156
*cpt1a*	0.71 ± 0.05 ^a^	0.59 ± 0.04 ^ab^	0.58 ± 0.05 ^ab^	0.53 ± 0.05 ^b^	**0.050**
*hfabp*	45.2 ± 4.15	51.7 ± 3.54	53.4 ± 4.99	43.1 ± 1.87	0.179
*cs*	0.51 ± 0.03 ^a^	0.48 ± 0.03 ^ab^	0.46 ± 0.03 ^ab^	0.41 ± 0.03 ^b^	**0.015**
*sirt1*	0.06 ± 0.01	0.06 ± 0.01	0.07 ± 0.01	0.06 ± 0.00	0.711
*sirt2*	0.16 ± 0.01	0.15 ± 0.01	0.15 ± 0.01	0.14 ± 0.01	0.489
*ucp1*	9.27 ± 0.94	11.0 ± 0.92	8.59 ± 0.74	8.35 ± 0.70	0.123
*gpx1*	1.29 ± 0.07	1.15 ± 0.06	1.26 ± 0.11	1.19 ± 0.11	0.674
*gpx4*	9.74 ± 1.14	11.4 ± 1.00	9.77 ± 1.00	8.48 ± 0.82	0.123
*prdx3*	0.75 ± 0.06	0.87 ± 0.06	0.88 ± 0.07	0.76 ± 0.07	0.309
*prdx5*	0.73 ± 0.06	0.69 ± 0.04	0.73 ± 0.07	0.64 ± 0.06	0.652
*cu-zn-sod/sod1*	3.53 ± 0.23	3.73 ± 0.18	3.80 ± 0.30	3.36 ± 0.19	0.516
*mn-sod/sod2*	0.83 ± 0.06 ^a^	1.13 ± 0.05 ^b^	1.04 ± 0.10 ^ab^	0.95 ± 0.09 ^ab^	**0.050**
*grp170*	1.00 ± 0.12	1.03 ± 0.09	1.03 ± 0.17	0.80 ± 0.09	0.509
*grp94*	3.51 ± 0.50	4.65 ± 0.39	4.05 ± 0.71	3.72 ± 0.42	0.442
*grp75*	0.47 ± 0.04	0.45 ± 0.03	0.46 ± 0.04	0.43 ± 0.04	0.893
*ctsb*	1.75 ± 0.17	1.55 ± 0.09	1.46 ± 0.08	1.38 ± 0.06	0.099
*ctsd*	1.07 ± 0.08	1.21 ± 0.12	0.95 ± 0.11	1.03 ± 0.09	0.344
*ctsl*	5.44 ± 0.56	6.14 ± 0.33	5.83 ± 0.47	5.35 ± 0.49	0.618

**Table 6:** Relative gene expression of head kidney in juvenile fish fed experimental diets. Gene complete names and functions can be found in Table S3. Values are the mean ± SEM of 10–12 fish. *p*-values from one-way ANOVA. Tukey post hoc test was used to identify differences between diet groups. Different lowercase letters stand for significant (*p* ≤ 0.05) differences between diet groups. All data values are in reference to the expression level of *il10* of fish fed CTRL diet with an arbitrary assigned value of 1.

	CTRL	NOPAP	MIX	PAP	*p*-Value
*il1β*	0.38 ± 0.08 ^a^	0.39 ± 0.04 ^a^	0.62 ± 0.10 ^b^	0.77 ± 0.09 ^c^	**0.005**
*il6*	0.07 ± 0.01	0.08 ± 0.02	0.07 ± 0.01	0.09 ± 0.02	0.170
*il7*	1.01 ± 0.11	1.02 ± 0.08	0.99 ± 0.08	1.18 ± 0.08	0.460
*il8*	0.09 ± 0.01 ^a^	0.22 ± 0.03 ^b^	0.22 ± 0.02 ^b^	0.25 ± 0.05 ^b^	**<0.001**
*il10*	1.01 ± 0.04	1.22 ± 0.10	1.26 ± 0.11	1.09 ± 0.08	0.233
*il12*	0.09 ± 0.01	0.09 ± 0.01	0.10 ± 0.01	0.10 ± 0.01	0.622
*il15*	0.34 ± 0.04	0.34 ± 0.02	0.32 ± 0.02	0.36 ± 0.04	0.867
*il34*	3.49 ± 0.31	3.31 ± 0.26	3.04 ± 0.09	3.57 ± 0.16	0.401
*tnfα*	0.24 ± 0.02 ^a^	0.28 ± 0.02 ^ab^	0.29 ± 0.02 ^ab^	0.34 ± 0.04 ^b^	**0.030**
*ccr3*	7.02 ± 0.62 ^a^	8.26 ± 0.62 ^ab^	7.51 ± 0.57 ^ab^	9.21 ± 0.39 ^b^	**0.044**
*ck8/ccl20*	1.57 ± 0.15 ^a^	1.96 ± 0.19 ^ab^	2.14 ± 0.26 ^ab^	2.39 ± 0.33 ^b^	**0.027**
*igm*	264.3 ± 32.5	295.2 ± 28.0	230.6 ± 21.4	314.2 ± 28	0.175
*igt-m*	3.47 ± 0.58 ^a^	3.44 ± 0.39 ^a^	2.77 ± 0.34 ^b^	1.92 ± 0.32 ^c^	**0.048**
*a2m*	0.11 ± 0.01	0.13 ± 0.03	0.12 ± 0.02	0.15 ± 0.03	0.300
*b2m*	143.8 ± 6.8	159.6 ± 11.8	133.1 ± 8.97	136.6 ± 14.7	0.359
*c3*	0.003 ± 0.001	0.004 ± 0.001	0.003 ± 0.001	0.003 ± 0.001	0.450
*casp3*	0.96 ± 0.05	1.18 ± 0.06	1.01 ± 0.06	1.10 ± 0.08	0.242
*cd3*	3.33 ± 0.20 ^a^	3.92 ± 0.28 ^ab^	3.59 ± 0.41 ^ab^	4.63 ± 0.41 ^b^	**0.049**
*cd4*	1.08 ± 0.09 ^a^	1.37 ± 0.10 ^ab^	1.34 ± 0.16 ^ab^	1.73 ± 0.15 ^b^	**0.008**
*cd8α*	1.58 ± 0.09 ^a^	1.93 ± 0.23 ^ab^	1.82 ± 0.21 ^ab^	2.27 ± 0.23 ^b^	**0.024**
*cd8β*	0.43 ± 0.04	0.54 ± 0.06	0.47 ± 0.07	0.56 ± 0.06	0.322
*zap70*	2.33 ± 0.20	2.84 ± 0.26	2.69 ± 0.31	2.85 ± 0.30	0.495
*csf1r1*	3.04 ± 0.25	3.42 ± 0.28	3.78 ± 0.51	3.20 ± 0.21	0.452
*mrc1*	13.83 ± 1.27	16.44 ± 2.19	13.71 ± 0.79	15.00 ± 1.02	0.725
*tlr2*	4.90 ± 0.37	6.15 ± 0.42	6.08 ± 0.52	5.86 ± 0.42	0.172
*tlr5*	1.17 ± 0.08	1.06 ± 0.06	1.02 ± 0.02	1.07 ± 0.07	0.473
*tlr9*	1.86 ± 0.19	2.18 ± 0.23	2.36 ± 0.30	2.45 ± 0.15	0.266
*clec10a*	2.48 ± 0.56	2.49 ± 0.55	2.20 ± 0.52	2.93 ± 0.72	0.950
*fcl*	0.06 ± 0.01	0.10 ± 0.02	0.06 ± 0.01	0.06 ± 0.01	0.286

## Data Availability

The original contributions presented in the study are included in the article/supplementary material; further inquiries can be directed to the corresponding author/s.

## Supplementary Materials

The following supporting information can be downloaded at: https://www.mdpi.com/article/10.3390/ani14152166/s1, Table S1: Detailed amino acid profile and summarized fatty acid composition of the different dietary groups; Table S2: PCR-array layout for hepatic gene expression profiling; Table S3: PCR-array layout for head kidney gene expression profiling; Table S4: Primers for qPCR amplification of hepatic genes; Table S5: Primers for qPCR amplification of head kidney genes.
