# Supplementary material for: Sustainable Fish Meal-Free Diets for Gilthead Sea Bream (Sparus aurata): Integrated Biomarker Response to Assess the Effects on Growth Performance, Lipid Metabolism, Antioxidant Defense and Immunological Status
Source: Animals (Basel). 2024 Jul 25;14(15):2166. doi: 10.3390/ani14152166 (PMC11311052; doi:10.3390/ani14152166)
Supplement: Supplementary file 1 [file animals-14-02166-s001.zip › Table S1 diets.pdf]

Table S1. Detailed amino acid profile and summarized fatty acid composition of the different dietary groups. Data are the mean ± SD from 2 replicates.

	CTRL	NOPAP	MIX	PAP
**Amino acids (*g/100 g dry feed*)**				
Arginine	35.7 ± 1.8	31.6 ± 0.9	34.6 ± 1.3	33.6 ± 1.5
Histidine	10.5 ± 0.8	10.9 ± 0.2	11.6 ± 0.5	11.2 ± 0.5
Lysine	29.8 ± 0.8	22.8 ± 1.8	29.2 ± 1.1	29.4 ± 1.7
Threonine	18.2 ± 0.6	18.1 ± 0.5	19.2 ± 0.4	17.3 ± 0.3
Isoleucine	18.5 ± 0.1	17.9 ± 0.3	17.2 ± 0.7	16.1 ± 0.3
Leucine	40.2 ± 0.1	41.7 ± 1.5	33.8 ± 1.1	34.3 ± 0.5
Valine	19.8 ± 0.3	20.4 ± 0.1	23.7 ± 0.4	21.5 ± 0.1
Methionine	12.2 ± 0.8	14.4 ± 0.5	14.4 ± 0.1	13.9 ± 0.5
Phenylalanine	22.9 ± 0.9	24.1 ± 0.4	20.8 ± 1.6	19.9 ± 1.1
Cystine	3.8 ± 0.3	3.5 ± 0.2	3.8 ± 0.2	3.6 ± 0.2
Tyrosine	20.4 ± 0.5	23.0 ± 1.6	20.3 ± 0.7	17.5 ± 0.1
Aspartic acid + Asparagine	35.5 ± 2.6	28.1 ± 1.7	31.5 ± 1.5	31.4 ± 0.6
Glutamic acid + Glutamine	84.7 ± 1.8	75.4 ± 2.0	55.5 ± 0.8	59.6 ± 3.4
Alanine	24.1 ± 0.3	26.0 ± 0.5	26.6 ± 1.3	26.8 ± 1.3
Glycine	23.1 ± 0.5	21.0 ± 0.7	27.0 ± 0.9	28.4 ± 0.9
Proline	28.2 ± 0.0	28.7 ± 0.6	24.3 ± 0.5	24.8 ± 1.2
Serine	24.0 ± 1.2	22.6 ± 0.3	23.2 ± 0.9	22.2 ± 0.6
Taurine	1.3 ± 0.0	6.5 ± 0.0	7.0 ± 0.2	7.3 ± 0.3
**Fatty acids (*% DM*)**				
Total saturated	2.02 ± 0.03	1.82 ± 0.03	2.23 ± 0.12	2.40 ± 0.03
Total monounsaturated	4.83 ± 0.05	5.12 ± 0.51	4.37 ± 0.36	4.68 ± 0.16
Total n-6 PUFA	2.11 ± 0.02	2.36 ± 0.12	1.90 ± 0.10	2.12 ± 0.05
Total n-3 PUFA	2.03 ± 0.04	1.18 ± 0.02	1.12 ± 0.04	1.16 ± 0.02
Total PUFA	4.14 ± 0.06	3.54 ± 0.14	3.02 ± 0.07	3.29 ± 0.07
