# Supplementary material for: Sustainable Fish Meal-Free Diets for Gilthead Sea Bream (Sparus aurata): Integrated Biomarker Response to Assess the Effects on Growth Performance, Lipid Metabolism, Antioxidant Defense and Immunological Status
Source: Animals (Basel). 2024 Jul 25;14(15):2166. doi: 10.3390/ani14152166 (PMC11311052; doi:10.3390/ani14152166)
Supplement: Supplementary file 1 [file animals-14-02166-s001.zip › Table S2 Liver PCR-array.pdf]

Table S2. PCR-array layout for hepatic gene expression profiling.

Function	Gene	Symbol	GenBank
Growth performance GH/IGF System	Growth hormone receptor-type 1	*ghr1*	AF438176
	Growth hormone receptor-type 2	*ghr2*	AY573601
	Insulin-like growth factor-I	*igf1*	AY996779
	Insulin-like growth factor-II	*igf2*	AY996778
	Insulin-like growth factor binding protein 1a	*igfbp1a*	KM522771
	Insulin-like growth factor binding protein 1b	*igfbp1b*	MH577189
	Insulin-like growth factor binding protein 2a	*igfbp2a*	MH577190
	Insulin-like growth factor binding protein 2b	*igfbp2b*	AF377998
	Insulin-like growth factor binding protein 4	*igfbp4*	KM658998
Lipid Metabolism	Elongation of very long chain fatty acids 1	*elovl1*	JX975700
	Elongation of very long chain fatty acids 4	*elovl4*	JX975701
	Elongation of very long chain fatty acids 5	*elovl5*	AY660879
	Elongation of very long chain fatty acids 6	*elovl6*	JX975702
	Fatty acid desaturase 2	*fads2*	AY055749
	Stearoyl-CoA desaturase 1a	*scd1a*	JQ277703
	Stearoyl-CoA desaturase 1b	*scd1b*	JQ277704
	Hepatic lipase	*hl*	EU254479
	Adipose triglyceride lipase	*atgl*	JX975711
	Lipoprotein lipase	*lpl*	AY495672
	85kDa calcium-independent phospholipase A2	*pla2g6*	JX975708
	Cholesterol 7-alpha-monooxygenase	*cyp7a1*	KX122017
	Peroxisome proliferator-activated receptor α	*pparα*	AY590299
	Peroxisome proliferator-activated receptor β	*pparβ*	AY590301
	Peroxisome proliferator-activated receptor γ	*pparγ*	AY590304
Energy Metabolism	Carnitine palmitoyltransferase 1A	*cpt1a*	JQ308822
	Fatty acid binding protein, heart	*hfabp*	JQ308834
	Citrate synthase	*cs*	JX975229
	Sirtuin1	*sirt1*	KF018666
	Sirtuin2	*sirt2*	KF018667
	Uncoupling protein 1	*ucp1*	FJ710211
Antioxidant Defence	Glutathione peroxidase 1	*gpx1*	DQ524992
	Glutathione peroxidase 4	*gpx4*	AM977818
	Peroxiredoxin 3	*prdx3*	GQ252681
	Peroxiredoxin 5	*prdx5*	GQ252683
	Superoxide dismutase [Cu-Zn]	*cu-zn-sod/sod1*	JQ308832
	Superoxide dismutase [Mn]	*mn-sod/sod2*	JQ308833
	Glucose-regulated protein, 170 kDa	*grp170*	JQ308821

	Glucose-regulated protein, 94 kDa	*grp94*	JQ308820
	Glucose-regulated protein, 75 kDa	*grp75*	DQ524993
Intracellular Proteolysis	Cathepsin B	*ctsb*	KJ524457
	Cathepsin D	*ctsd*	AF036319
	Cathepsin L	*ctsl*	KM522787
