# Supplementary material for: Sustainable Fish Meal-Free Diets for Gilthead Sea Bream (Sparus aurata): Integrated Biomarker Response to Assess the Effects on Growth Performance, Lipid Metabolism, Antioxidant Defense and Immunological Status
Source: Animals (Basel). 2024 Jul 25;14(15):2166. doi: 10.3390/ani14152166 (PMC11311052; doi:10.3390/ani14152166)
Supplement: Supplementary file 1 [file animals-14-02166-s001.zip › Table S3 HK PCR-array.pdf]

Table S3. PCR-array layout for head kidney gene expression profiling.

Function	Gene	Symbol	GenBank
Cytokines and related proteins	Interleukin-1 beta	*il1β*	AJ419178
	Interleukin-6	*il6*	EU244588
	Interleukin-7	*il7*	JX976618
	Interleukin-8	*il8*	JX976619
	Interleukin-10	*il10*	JX976621
	Interleukin 12 subunit beta	*il12*	JX976624
	Interleukin-15	*il15*	JX976625
	Interleukin-34	*il34*	JX976629
	Tumor necrosis factor-alpha	*tnfα*	AJ413189
	C-C chemokine receptor type 3	*ccr3*	KF857317
	C-C chemokine CK8/C-C motif chemokine 20	*ck8/ccl20*	GU181393
Adaptive immunity	Immunoglobulin M	*igm*	JQ811851
	Immunoglobulin T membrane-bound form	*igt-m*	KX599201
Acute phase response proteins and Proteolytic activity	Alpha-2-macroglobulin	*a2m*	AY358020
	Beta-2-microglobulin	*b2m*	MF979881
	Complement factor C3	*c3*	HM543456
	Caspase 3	*casp3*	EU722334
T cell markers & Monocyte/macrophage markers	Cluster of differentiation 3 zeta chain	*cd3*	MF175235
	Cluster of differentiation 4–1	*cd4*	AM489485
	Cluster of differentiation 8 alpha	*cd8α*	EU921630
	Cluster of differentiation 8 beta	*cd8β*	KX231275
	Zeta-chain-associated protein kinase 70	*zap70*	MF175239
	Macrophage colony-stimulating factor 1 receptor 1	*csf1r1*	AM050293
	Macrophage mannose receptor 1	*mrc1*	KF857326
Pathogen associated microbial pattern (PAMP)	Toll-like receptor 2	*tlr2*	KF857323
	Toll-like receptor 5	*tlr5*	KF857324
	Toll-like receptor 9	*tlr9*	AY751797
	C-type lectin domain family 10 member A	*clec10a*	KF857329
	Fucolectin	*fcl*	KF857331
