# Supplementary material for: Sustainable Fish Meal-Free Diets for Gilthead Sea Bream (Sparus aurata): Integrated Biomarker Response to Assess the Effects on Growth Performance, Lipid Metabolism, Antioxidant Defense and Immunological Status
Source: Animals (Basel). 2024 Jul 25;14(15):2166. doi: 10.3390/ani14152166 (PMC11311052; doi:10.3390/ani14152166)
Supplement: Supplementary file 1 [file animals-14-02166-s001.zip › Table S4 liver primers-revised.pdf]

Supplementary Table S4. Primers for qPCR amplification of hepatic genes.

Gene	Symbol	Genbank	Sequence	Tm	Ta	Primers efficiency (%)
Growth hormone receptor I	*ghr1*	AF438176	F: ACC TGT CAG CCA CCA CAT GA<br>R: TCG TGC AGA TCT GGG TCG TA	67<br>66	61	97
Growth hormone receptor II	*ghr2*	AY573601	F: GAG TGA ACC CGG CCT GAC AG<br>R: GCG GTG GTA TCT GAT TCA TGG T	69<br>64	59	98
Insulin-like growth factor-I	*igf1*	AY996779	F: TGT CTA GCG CTC TTT CCT TTC A<br>R: AGA GGG TGT GGC TAC AGG AGA TAC	67<br>67	58	95
Insulin-like growth factor-II	*igf2i*	AY996778	F: TGG GAT CGT AGA GGA GTG TTG T<br>R: CTG TAG AGA GGT GGC CGA CA	65<br>66	60	93
Insulin-like growth factor binding protein 1a	*igfbp1a*	KM522771	F: ACA AAC CAA AAC AGT GCG AGT CCT C<br>R: CCG TTC CAA GAG TTC ACA CAC CAG	67<br>67	62	98
Insulin-like growth factor binding protein 1b	*igfbp1b*	MH577189	F: GCC AAA CAG TGT GAG TCA TC<br>R: ATC TTC TTC CCG TTC CAG G	63<br>64	58	96
Insulin-like growth factor binding protein 2a	*igfbp2a*	MH577190	F: CCA GCA AAG AGA CCA CCT<br>R: TCT TCA TCT CCT GCC TGT G	62<br>62	57	97
Insulin-like growth factor binding protein 2b	*igfbp2b*	AF377998	F: AGC GAT GTG TCC TGA GAT AGT GAG<br>R: GCA CCG TGG CGT GTA GAC C	65<br>70	60	95
Insulin-like growth factor binding protein 4	*igfbp4*	KM658998	F: GGC ATC AAA CAC CCG CAC AC<br>R: ATC CAC GCA CCA GCA CTT CC	68<br>68	63	100
Elongation of very long chain fatty acids 1	*elovl1*	JX975700	F: CTT CCT ACA CAT CTT CCA CCA CTC<br>R: CCA TTC CAC CAG GAG CAA AGG	64<br>66	59	98
Elongation of very long chain fatty acids 4	*elovl4*	JX975701	F: CGG TGG CAA TCA TCT TCC<br>R: TCA ACT GGC TGT CTG TGT	63<br>63	58	94
Elongation of very long chain fatty acids 5	*elovl5*	AY660879	F: CCT CCT GGT GCT CT ACA AT<br>R: GTG AGT GTC CTG GCA GTA	63<br>64	58	96
Elongation of very long chain fatty acids 6	*elovl6*	JX975702	F: GTG CTG CTC TAC TCC TGG TA<br>R: ACG GCA TGG ACC AAG TAG T	64<br>64	59	95
Fatty acid desaturase 2	*fads2*	AY055749	F: GCA GGC GGA GAG CGA CGG TCT GTT CC<br>R: AGC AGG ATG TGA CCC AGG TGG AGG CAG AAG	69<br>68	63	97
Stearoyl-CoA desaturase 1a	*scd1a*	JQ277703	F: CGG AGG CGG AGG CGT TGG AGA AGA AG<br>R: AGG GAG ACG GCG TAC AGG GCA CCT ATA TG	68<br>68	63	92
Stearoyl-CoA desaturase 1b	*scd1b*	JQ277704	F: GCT CAA TCT CAC CAC CGC CTT CAT AG<br>R: GCT GCC GTC GCC CGT TCT CTG	67<br>71	62	99
Hepatic lipase	*hl*	EU254479	F: TTG TAG AAG GTG AGG AAA ACT G<br>R: GCT CTC CAT CAG ACC ATC C	61<br>66	59	94

Lipoprotein lipase	*lpl*	AY495672	F: CGT TGC CAA GTT TGT GAC CTG<br>R: AGG GTG TTC TGG TTG TCT GC	64<br>65	59	98
Adipose triglyceride lipase	*atgl*	JX975711	F: GTG CTT CAG TCC TGG ATG TCT TC<br>R: AGC CTT GCA GGT CCA TGT TGA	65<br>67	60	94
85kDa calcium-independent phospholipase A2	*pla2g6*	JX975708	F: CGC CAA GGA ACT CGG AAA GAT GCT<br>R: ACC GCA CAG CCA TCA GAG TCT	67<br>68	62	95
Cholesterol 7-alpha-monooxygenase	*cyp7a1*	KX122017	F: CCC TGC TAT TAA AGT CCC ACC TCT<br>R: ATC GTA GGT AGG CTG GAG GAT TC	65<br>65	60	97
Peroxisome proliferator-activated receptor α	*pparα*	AY590299	F: TCT CTT CAG CCC ACC ATC CC<br>R: ATC CCA GCG TGT CGT CTC C	67<br>68	62	96
Peroxisome proliferator-activated receptor β	*pparβ*	AY590301	F: AGG CGA GGG AGA GTG AGG ATG AGG AG<br>R: CTG TTC TGA AAG CGA GGG TGA CGA TGT TTG	68<br>66	62	99
Peroxisomeproliferator-activated receptor γ	*pparγ*	AY590304	F: CGC CGT GGA CCT GTC AGA GC<br>R: GGA ATG GAT GGA GGA GGA GGA GAT GG	70<br>68	63	93
Carnitine palmitoyltransferase 1A	*cpt1a*	JQ308822	F: GTG CCT TCG TTC GTT CCA TGA TC<br>R: TGA TGC TTA TCT GCT GCC TGT TTG	66<br>65	60	92
Fatty acid binding protein, heart	*hfabp*	JQ308834	F: CTG GGT GTG GGC TTC GCT AC<br>R: CTC TGT GTT CTT GAT GGT GCT CTG	69<br>65	60	100
Citrate synthase	*cs*	JX975229	F: TCC AGG AGG TGA CGA GCC<br>R: GTG ACC AGC AGC CAG AAG AG	68<br>66	61	95
Sirtuin1	*sirt1*	KF018666	F: GGT TCC TAC AGT TTC ATC CAG CAG CAC ATC<br>R: CCT CAG AAT GGT CCT CGG ATC GGT CTC	68<br>70	63	97
Sirtuin2	*sirt2*	KF018667	F: GAA CAA TCC GAC GAC AGC AGT GAA G<br>R: AGG TTA CGC AGG AAG TCC ATC TCT	67<br>67	62	92
Uncoupling protein 1	*ucp1*	FJ710211	F: GCA CAC TAC CCA ACA TCA CAA G<br>R: CGC CGA ACG CAG AAA CAA AG	63<br>65	58	99
Glutathione peroxidase 1	*gpx1*	DQ524992	F: GAA GGT GGA TGT GAA TGG AAA AGA TG<br>R: CTG ACG GGA CTC CAA ATG ATG G	63<br>65	58	96
Glutathione peroxidase 4	*gpx4*	AM977818	F: TGC GTC TGA TAG GGT CCA CTG TC<br>R: GTC TGC CAG TCC TCT GTC GG	67<br>67	62	92
Peroxiredoxin 3	*prdx3*	GQ252681	F: ATC AAC ACC CCA CGC AAG ACT G<br>R: ACC GTT TGG ATC AAT GAG GAA CAG ACC	67<br>67	62	92
Peroxiredoxin 5	*prdx5*	GQ252683	F: GAG CAC GGA ACA GAT GGC AAG G<br>R: TCC ACA TTG ATC TTC TTC ACG ACT CC	69<br>65	60	92
Superoxide dismutase [Cu-Zn]	*cu-zn-sod / sod1*	JQ308832	F: TCA CGG ACA AGA TGC TCA CTC TC<br>R: GGT TCT GCC AAT GAT GGA CAA GG	66<br>66	61	92
Superoxide dismutase [Mn]	*mn-sod / sod2*	JQ308833	F: CCT GAC CTG ACC TAC GAC TAT GG	66	60	93

			R: AGT GCC TCC TGA TAT TTC TCC TCT G	65		
Glucose-regulated protein, 170 kDa	*grp170*	JQ308821	F: CAG AGG AGG CAG ACA GCA AGA C<br>R: TTC TCA GAC TCA GCA TTT CCA GAT TTC	68<br>63	58	92
Glucose-regulated protein, 94 kDa	*grp-94*	JQ308820	F: AAG GCA CAG GCT TAC CAG ACA G<br>R: CTT CAG CAT CAT CGC CGA CTT TC	67<br>66	61	96
Glucose-regulated protein, 75 kDa	*grp-75*	DQ524993	F: TCC GGT GTG GAT CTG ACC AAA GAC<br>R: TGT TTA GGC CCA GAA GCA TCC ATG	68<br>67	62	99
Cathepsin B	*ctsb*	KJ524457	F: TGA TTC CCA TGT CGG TTG TC<br>R: GGG TCT ACT GCC ATT CAC AT	63<br>63	58	92
Cathepsin D	*ctsd*	AF036319	F: CAC ACT GGG AGA CCT GCA CTA TGT CAA TG<br>R: ATT GCC AAC TTG AAG TCC GTC CAT ACC	69<br>67	62	93
Cathepsin L	*ctsl*	KM522787	F: GGG AAC GGA TGA CCA GCC TTG T<br>R: CGG TGT CAT TGG CAG AGT TGT AGT TG	70<br>67	62	95
Beta-actin	*actb*	KY388508	F: TCC TGC GGA ATC CAT GAG A<br>R: GAC GTC GCA CTT CAT GAT GCT	63<br>65	58	99
