# Supplementary material for: Sustainable Fish Meal-Free Diets for Gilthead Sea Bream (Sparus aurata): Integrated Biomarker Response to Assess the Effects on Growth Performance, Lipid Metabolism, Antioxidant Defense and Immunological Status
Source: Animals (Basel). 2024 Jul 25;14(15):2166. doi: 10.3390/ani14152166 (PMC11311052; doi:10.3390/ani14152166)
Supplement: Supplementary file 1 [file animals-14-02166-s001.zip › Table S5 HK primers-revised.pdf]

Supplementary Table S5. Primers for qPCR amplification of head kidney genes.

Gene	Symbol	Genbank	Sequence	Tm	Ta	Primers efficiency (%)
Interleukin-1 beta	*il1β*	AJ419178	F: GCG ACC TAC CTG CCA CCT ACA CC<br>R: TCG TCC ACC GCC TCC AGA TGC	68<br>68	62	96
Interleukin-6	*il6*	EU244588	F: TCT TGA AGG TGG TGC TGG AAG TG<br>R: AAG GAC AAT CTG CTG GAA GTG AGG	64<br>63	58	92
Interleukin-7	*il7*	JX976618	F: CTA TCT CTG TCC CTG TCC TGT GA<br>R: TGC GGA TGG TTG CCT TGT AAT	66<br>65	60	100
Interleukin-8	*il8*	JX976619	F: CAG CAG AGT CTT CAT CGT CAC TAT TG<br>R: AGG CTC GCT TCA CTG ATG G	65<br>66	60	99
Interleukin-10	*il10*	JX976621	F: AAC ATC CTG GGC TTC TAT CTG<br>R: GTG TCC TCC GTC TCA TCT G	62<br>63	57	99
Interleukin 12 subunit beta	*il12*	JX976624	F: ATT CCC TGT GTG GTG GCT GCT<br>R: GCT GGC ATC CTG GCA CTG AAT	68<br>67	62	100
Interleukin-15	*il15*	JX976625	F: GAG ACC AGC GAG CGA AAG GCA TCC<br>R: GCC AGA ACA GGT TAC AGG TTG ACA GGA A	70<br>68	63	98
Interleukin-34	*il34*	JX976629	F: TCT GTC TGC CTG CTG GTA G<br>R: ATG CTG GCT GGT GTC TGG	64<br>66	59	99
Tumor necrosis factor-alpha	*tnfα*	AJ413189	F: CAG GCG TCG TTC AGA GTC TC<br>R: CTG TGG CTG AGA GCT GTG AG	65<br>66	60	100
C-C chemokine receptor type 3	*ccr3*	KF857317	F: CTA CAT CAG CAT CAC CAT ACG CAT CCT<br>R: TGG CAC GGC ACT TCT CCT TCA	67<br>69	62	95
C-C chemokine CK8 / C-C motif chemokine 20	*ck8 / ccl20*	GU181393	F: CCG TCC TCA TCT GCT TCA TAC T<br>R: GCT CTG CCG TTG ATG GAA C	64<br>64	59	97
Immunoglobulin M	*igm*	JQ811851	F: ACC TCA GCG TCC TTC AGT GTT TAT GAT GCC<br>R: CAG CGT CGT CGT CAA CAA GCC AAG C	68<br>70	63	94
Immunoglobulin T membrane-bound form	*igt-m*	KX599201	F: AGA CGA TGC CAG TGA AGA GGA TGA GT<br>R: CGA AGG AGG AGG CTG TGG ACC A	67<br>69	62	98
Alpha-2-macroglobulin	*a2m*	AY358020	F: TCC TGG GTG ACA TTC TGG GT<br>R: CCG TAT GGC ATC CTC AGC AG	66<br>66	61	95
Beta 2 microglobulin	*b2m*	MF979881	F: GGC ACT TCC ATC TGA CCA AGA<br>R: GCT GAA CCG CTC TCC ACG	65<br>67	60	99
Complement factor C3	*c3*	HM543456	F: GCT TAC GCT CTT CTT GCT CTG GTG AA<br>R: CAT CTG ACA ACT GGT CTG GCA TCG T	67<br>68	62	96
Caspase 3	*casp3*	EU722334	F: GCC AAC GGA CCT GGA CCT G<br>R: CCA TCG CCT CTC CTC GCA TCT A	68<br>67	62	100
Cluster of differentiation 3 zeta chain	*cd3*	MF175235	F: ATG GCG GTC CAG ACG AGG GTT TC<br>R: ACC AGC GAG GAC AGG ACC AGC AG	69<br>71	63	96
			F: TCC TCC TCC TCG TCC TCG TT	68	61	97

Cluster of differentiation 4–1	*cd4*	AM489485	R: GGT GTC TCA TCT TCC GCT GTC T	66		
Cluster of differentiation 8 alpha	*cd8α*	EU921630	F: GCA GCA ACG GTA ACA CGA ACG<br>R: CCA GTA TGA GCG GAG TAC AGA ACA	67<br>65	60	92
Cluster of differentiation 8 beta	*cd8β*	KX231275	F: CCG AAA TGT GGA AGA CTG GAA CTC<br>R: CTT TGG AGG TAA GGT TGG AGG GAT	65<br>66	60	98
Zeta-chain-associated protein kinase 70	*zap70*	MF175239	F: TGG TGA AGG AGG AGA TGA TGA GG<br>R: GCG AAC GAT GTA GCG GTT GT	65<br>66	60	100
Macrophage colony-stimulating factor 1 receptor 1	*csf1r1*	AM050293	F: TTG CGT GTG GTG AGG AAG GAA GGT<br>R: AGC AGG CAG GGC AGC AGG TA	68<br>70	63	98
Macrophage mannose receptor 1	*mrc1*	KF857326	F: CTT CCG ACC GTA CCT GTA CCT ACT CA<br>R: CGA TTC CAG CCT TCC GCA CAC TTA	67<br>68	62	92
Toll-like receptor 2	*tlr2*	KF857323	F: CAT CTG CGA CTC TCC TCT CTT CCT<br>R: ATT CAA CAA TGG AGC GGT GGA CTT	67<br>66	61	100
Toll-like receptor 5	*tlr5*	KF857324	F: TCG CCA ATC TGA CGG ACC TGA G<br>R: CAG AAC GCC GAT GTG GTT GTA AGA C	69<br>67	62	92
Toll-like receptor 9	*tlr9*	AY751797	F: GCC TTC CTT GTC TGC TCT TTC T<br>R: GCC GTA GAG GTG CTT CAG TAG	64<br>65	59	99
C-type lectin domain family 10 member A	*clec10a*	KF857329	F: CGA CTC TGG ACT CCC TCA<br>R: CGT TGT TGA TGG TGC GTT C	64<br>62	58	95
Fucolectin	*fcl*	KF857331	F: CCA TAC TGC TGA ACA GAC CAA CC<br>R: TGA TGG AGG TGA CGA TGT AGG A	65<br>64	59	94
Beta-actin	*actb*	KY388508	F: TCC TGC GGA ATC CAT GAG A<br>R: GAC GTC GCA CTT CAT GAT GCT	63<br>65	58	99
